# Adding anthropometric measures of regional adiposity to BMI improves prediction of cardiometabolic, inflammatory and adipokines profiles in youths: a cross-sectional study

**DOI:** 10.1186/s12887-015-0486-5

**Published:** 2015-10-24

**Authors:** Hanen Samouda, Carine de Beaufort, Saverio Stranges, Benjamin C. Guinhouya, Georges Gilson, Marco Hirsch, Julien Jacobs, Sonia Leite, Michel Vaillant, Frédéric Dadoun

**Affiliations:** Population Health Department, Epidemiology and Public Health Research Unit, Luxembourg Institute of Health, 1A-B, rue Thomas Edison, L-1445 Strassen, Luxembourg; Centre Hospitalier de Luxembourg, Diabetes & Endocrinology Care Clinique Pédiatrique (DECCP), L-1210 Luxembourg, Luxembourg; Faculty for Health engineering and management, UDSL/ILIS, University Lille-Northern France, EA 2694, Laboratory of Public Health, F-59120 Loos, France; Department of Clinical Biology, Centre Hospitalier de Luxembourg, L-1210 Luxembourg, Luxembourg; ZithaKlinik, Rheumatology Department, L-2763 Luxembourg, Luxembourg; Luxembourg Institute of Health, Centre of Competence for Methodology and Statistics (CCMS), L-1445 Strassen, Luxembourg; Endocrinology and Diabetology Department, Centre Hospitalier de Luxembourg, L-1210 Luxembourg, Luxembourg

**Keywords:** Obesity, Overweight, Body mass index, Body fat distribution, DXA, Anthropometry

## Abstract

**Background:**

Paediatric research analysing the relationship between the easy-to-use anthropometric measures for adiposity and cardiometabolic risk factors remains highly controversial in youth. Several studies suggest that only body mass index (BMI), a measure of relative weight, constitutes an accurate predictor, whereas others highlight the potential role of waist-to-hip ratio (WHR), waist circumference (Waist C), and waist-to-height ratio (WHtR). In this study, we examined the effectiveness of adding anthropometric measures of body fat distribution (Waist C Z Score, WHR Z Score and/or WHtR) to BMI Z Score to predict cardiometabolic risk factors in overweight and obese youth. We also examined the consistency of these associations with the “total fat mass + trunk/legs fat mass” and/or the “total fat mass + trunk fat mass” combinations, as assessed by dual energy X-ray absorptiometry (DXA), the gold standard measurement of body composition.

**Methods:**

Anthropometric and DXA measurements of total and regional adiposity, as well as a comprehensive assessment of cardiometabolic, inflammatory and adipokines profiles were performed in 203 overweight and obese 7–17 year-old youths from the *Paediatrics Clinic, Centre Hospitalier* de Luxembourg.

**Results:**

Adding only one anthropometric surrogate of regional fat to BMI Z Score improved the prediction of insulin resistance (WHR Z Score, R^2^: 45.9 %. Waist C Z Score, R^2^: 45.5 %), HDL-cholesterol (WHR Z Score, R^2^: 9.6 %. Waist C Z Score, R^2^: 10.8 %. WHtR, R^2^: 6.5 %), triglycerides (WHR Z Score, R^2^: 11.7 %. Waist C Z Score, R^2^: 12.2 %), adiponectin (WHR Z Score, R^2^: 14.3 %. Waist C Z Score, R^2^: 17.7 %), CRP (WHR Z Score, R^2^: 18.2 %. WHtR, R^2^: 23.3 %), systolic (WHtR, R^2^: 22.4 %), diastolic blood pressure (WHtR, R^2^: 20 %) and fibrinogen (WHtR, R^2^: 21.8 %). Moreover, WHR Z Score, Waist C Z Score and/or WHtR showed an independent significant contribution according to these models. These results were in line with the DXA findings.

**Conclusions:**

Adding anthropometric measures of regional adiposity to BMI Z Score improves the prediction of cardiometabolic, inflammatory and adipokines profiles in youth.

## Background

Several studies have focused on the presence of early biological abnormalities in excess-weight youths, including elevated fasting glycaemia, insulin resistance, hypertriglyceridemia, high-density lipoprotein cholesterol (HDL-cholesterol), elevated blood pressure and causing several comorbidities in adults [1–5]. Furthermore, some adipokines, namely leptin, adiponectin and resistin, have been identified as potential risk markers for a systemic low-grade inflammation state, which might lead to insulin resistance, type-2 diabetes and cardiovascular (CV) diseases [6–8].

Moreover, beyond global excess weight, the role of the abdominovisceral adiposity as independent cardiometabolic risk factor has been underlined from children onwards [9], while more peripheral fat has been considered as protective [10].

Magnetic Resonance Imaging (MRI), Computed Tomography-Scan (CT-Scan) and Dual-energy X ray Absorptiometry (DXA) have been described as the gold standard of adiposity measurement and used to accurately assess body fat distribution and related comorbidities [9, 11, 12]. However, these techniques are still no accessible because of their high cost and irradiation in the case of CT-Scan measurements as well [11, 12].

Therefore, in order to assess the comorbidities associated with overweight and obesity and abdomino-visceral adiposity in youths, the identification of simple and accurate anthropometric methods that can be used with efficiency as clinical and research tools is essential.

Studies analysing the relationship between the easy-to-use anthropometric measures for total fat mass, body fat distribution and cardiometabolic risk factors are highly controversial when it comes to youths. Several authors suggested that only body mass index (BMI) constitutes an accurate predictor of biological abnormalities and cardiometabolic impairments [13–17], whereas others highlighted the role of the waist-to-hip ratio (WHR) [18, 19], waist circumference (Waist C) [20, 21] and/or waist-to-height ratio (WHtR) [22, 23]. Furthermore, certain studies showed no significant differences in the ability of BMI and WHR [24], BMI and Waist C [25], BMI and WHtR [26], as well as Waist C and WHtR [27] to predict cardiometabolic risk factors. Finally, in some other studies, differential associations were observed between CV risk factors and anthropometric measures [28, 29].

In adults, extensive studies showed that adding anthropometric measures of body fat distribution such as WHR or Waist C, to BMI, allows predicting CV risk factors, diseases and death more accurately [2, 30–34]. This type of associations has not really been developed in paediatric populations. Indeed, in an attempt to predict cardiometabolic risk factors in youths, some previous paediatric studies either tested the efficiency of a single anthropometric measurement [14, 21, 23, 25] or assessed the contribution of BMI only as a potential confounder of other variables involved [18, 20, 27, 29].

The present study investigated the ability of the “BMI and Waist C”, “BMI and WHR” and/or “BMI and WHtR” associations to predict cardiometabolic risk factors in overweight and obese youths. The consistency of our findings was evaluated by assessing the ability to predict the same risk factors presented by the associations between total fat mass and trunk fat mass, respectively total fat mass and trunk/legs fat mass as obtained by dual energy X-ray absorptiometry (DXA), which is the body-composition gold-standard analysis.

## Methods

### Participants

Two hundred three overweight and/or obese children (52.2 % female) according to the IOTF definition [35], aged 7 to17 years old, and visiting the *Diabetes & Endocrinology Care Paediatrics Clinic, Centre Hospitalier de Luxembourg*, were invited to participate in a cross-sectional study performed between September 2006 and June 2008. The parents gave their written informed consent. The study was approved by the National Ethics Committee and authorized by the National Commission for Data Protection in Luxembourg.

### Anthropometry and body composition

Height, weight, waist and hip circumferences have been performed according to the recommendations of Lohmann [36]. BMI, WHR and WHtR ratios were calculated. Total, trunk and legs fat masses were measured by DXA using the Hologic^®^QDR4500W densitometer. Trunk/legs fat mass index was calculated.

### Clinical and biological measurements

Blood pressures was measured with an aneroid sphygmomanometer (Welch AL) in the sitting position: 3 readings were performed and the average was retained. Systolic blood pressure (SBP) and diastolic blood pressure (DBP) Z Scores were calculated according to the formula proposed by The Fourth Report on the Diagnosis, Evaluation, and Treatment of High Blood Pressure in Children and Adolescents [37]*.* Roche reagents on a P module of Roche Modular (Basel, Switzerland) were used to assess fasting glucose, triglycerides, HDL-cholesterol and low-density lipoprotein cholesterol (LDL-cholesterol). An Olympus latex reagent was used on the same P module of a Roche Modular to measure C-reactive protein (CRP). A chimiluminescent assay on Siemens Immulite 2000 (Deerfield, USA) was used to measure fasting insulin. Fibrinogen was assessed on Stago Compact (Asnières sur Seine, France). ELISA kits provided by Mediagnost (Reutlingen, Germany) were used to assess leptin, adiponectin and resistin. We also calculated the homeostasis model assessment of insulin resistance [HOMA IR = fasting insulin (μU/ml) × fasting glucose (mmol/l)/22.5] [38] and the quantitative insulin sensitivity check index [QUICKI index = 1/(log fasting insulin in μU/ml + log glucose in mg/dl)] [39]. Tanner stages were assessed [40, 41].

### Statistical analyses

The combination of the Kolmogorov-Smirnov test and of the Lilliefors’ test was used to check the normal data distribution. Triglycerides, HDL cholesterol, fasting insulin, HOMA IR, CRP, fibrinogen, adiponectin, leptin and resistin were log transformed (skewed variables).

Mean ± sd and/or percentages were calculated and compared by the Student’s *t* test (descriptive data). In the absence of national L,M,S data, BMI, Waist C and WHR Z Scores, as well as the overweight (boys: ≥ 91th percentile; girls: ≥ 89th percentile) and obesity (≥99th percentile) thresholds, were defined according to the L, M, S Dutch values [42, 43] and the IOTF definition [35].

#### Anthropometric and DXA prediction of cardiometabolic risk factors

To test the ability of each single anthropometric variable to predict the risk factors, the univariate linear analysis [Pearson’s R] was used. To assess the combined effect of the “BMI and Waist C”, “BMI and WHR”, “BMI and WHtR” as well as “total and trunk fat masses” and “total and trunk/legs fat masses” associations on the prediction of the risk factors, multivariable linear analyses were performed. An additional effect of Waist C Z Score, WHR Z Score and/or WHtR was highlighted when 1.the global variance of the model (R^2^) was improved and 2.the variable showed an independent significant contribution to the model (significant r^2^_partial_), independently of the BMI Z Score. All models were age-, sex- and pubertal status adjusted. To test the consistency of the anthropometry and DXA findings, similar analyses were performed to assess the potential additional impact of the trunk fat mass and/or the trunk/legs fat index, beyond the total fat mass. Results with a *p*-value < 0.05 were considered statistically significant. Statistical analyses were performed using SPSS^®^ for Windows Version 17.0.

## Results

The anthropometric, body composition and biological characteristics of the participants are summarized in Table 1.

**Table 1:** Subject characteristics

	Girls	Boys	All children
N	106	97	203
Age (years)	12.2 ± 2.5	11.8 ± 2.4	12.0 ± 2.4
Pubertal status (Percentages)			
Yes	84 (79.2 %)	47 (48.5 %)	131 (64.5 %)
No	22 (20.8 %)	50 (51.5 %)	72 (35.5 %)
Anthropometry			
BMI (kg/m^2^)	28.5 ± 5.6	28.2 ± 4.9	28.3 ± 5.3
BMI Z score	2.42 ± 0.58	2.68 ± 0.53^*^	2.54 ± 0.57
Waist C (cm)	83.8 ± 12.4	86.5 ± 11.5	85.1 ± 12.0
Waist C Z score	2.22 ± 0.63	2.46 ± 0.58^*^	2.33 ± 0.62
WHtR^a^	0.54 ± 0.06	0.56 ± 0.05^*^	0.55 ± 0.06
WHR^b^	0.84 ± 0.06	0.89 ± 0.05^**^	0.86 ± 0.06
WHR Z score	0.71 ± 0.89	0.85 ± 0.95	0.78 ± 0.92
Biology			
Fasting glucose (mg/dl)	86.2 ± 6.8	86.9 ± 6.2	86.5 ± 6.5
Fasting insulin (mUI/l)	17.5 ± 8.5	14.8 ± 8.3^*^	16.2 ± 8.5
HOMA IR	3.76 ± 1.98	3.21 ± 1.87^*^	3.50 ± 1.94
QUICKI	0.321 ± 0.024	0.330 ± 0.027^*^	0.326 ± 0.026
Triglycerides (mg/dl)	98.4 ± 58.4	90.0 ± 51.1	94.3 ± 55.1
HDL cholesterol (mg/dl)	54.4 ± 12.7	52.9 ± 12.1	53.7 ± 12.4
LDL cholesterol (mg/dl)	92.3 ± 29.0	93.0 ± 28.2	92.6 ± 28.6
CRP (mg/l)	2.9 ± 4.1	3.2 ± 3.8	3.1 ± 4.0
Fibrinogen (g/l)	3.7 ± 0.7	3.6 ± 0.6	3.6 ± 0.7
Adiponectin (μg/ml)	8.0 ± 4.7	7.8 ± 4.5	7.9 ± 4.6
Leptin (ng/ml)	38.7 ± 23.1	27.4 ± 16.1^**^	33.3 ± 20.8
Resistin (ng/ml)	5.3 ± 2.2	5.1 ± 2.0	5.2 ± 2.1
DXA			
Total fat mass (kg)	32.51 ± 14.29	30.11 ± 10.85	31.37 ± 12.80
Trunk fat mass (kg)	15.07 ± 7.14	14.17 ± 5.87	14.64 ± 6.57
Trunk/legs fat mass index	1.22 ± 0.24	1.27 ± 0.28	1.24 ± 0.26
Blood pressure			
SBP (mmHg)	117 ± 12	118 ± 14	117 ± 13
SBP Z score	0.99 ± 1.04	0.91 ± 1.10	0.95 ± 1.07
DBP (mmHg)	71 ± 9	72 ± 8	72 ± 9
DBP Z score	0.75 ± 0.78	0.81 ± 0.64	0.78 ± 0.71

Data are N and/or means ± SD

^*^ *P*-value < 0.05; ^**^ *P*-value < 0.001 for sex difference

^a^WHtR (waist to height ratio)

^b^WHR (waist-to-hip ratio)

### Relationships between single anthropometric variables and CV risk factors

BMI Z Score was the most accurate single predictor of fasting glucose, fasting insulin, HOMA IR, QUICKI, leptin and resistin. Triglycerides and HDL cholesterol were most accurately predicted by Waist C Z Score. Blood pressure, CRP and fibrinogen were most accurately predicted by WHtR. WHR Z Score was the most accurate single predictor of adiponectin (Table 2).

**Table 2:** Relationships between a single anthropometric measurement and biological variables

Variable	BMI Z score	Waist C Z score	WHtR	WHR Z score
Pearson’s R				
Fasting glucose	0.235^*^	0.176^*^	0.193^*^	0.057
Fasting insulin^a^	0.490^**^	0.483^**^	0.463^**^	0.295^**^
HOMA IR^a^	0.493^**^	0.480^**^	0.463^**^	0.290^**^
QUICKI	−0.475^**^	−0.463^**^	−0.444^**^	−0.283^**^
Triglycerides^a^	0.205^*^	0.270^**^	0.250^**^	0.249^**^
HDL cholesterol^a^	−0.205^*^	−0.293^**^	−0.252^**^	−0.273^**^
LDL cholesterol	−0.047	−0.013	0.003	0.018
SBP Z score	0.385^**^	0.389^**^	0.433^**^	0.198^*^
DBP Z score	0.392^**^	0.353^**^	0.418^**^	0.186^*^
CRP^a^	0.374^**^	0.388^**^	0.472^**^	0.261^**^
Fibrinogen^a^	0.341^**^	0.316^**^	0.375^**^	0.193^*^
Adiponectin^a^	−0.187^*^	−0.277^**^	−0.201^*^	−0.279^**^
Leptin^a^	0.551^**^	0.498^**^	0.546^**^	0.119
Resistin^a^	0.229^*^	0.181^*^	0.191^*^	0.064

Data are Pearson’s R (univariate linear analysis) for single biological variables

^*^ *P*-value < 0.05; ^**^ *P*-value < 0.001

^a^Log-transformed variables

### Prediction of CV risk factors using models adding anthropometric surrogates of body fat distribution to general adiposity measurements

The initial model including BMI Z Score, age, sex and pubertal status accounted for respectively 7.4, 43.7, 42.7, 41.4, 7.9, 4.3, 18.8, 17.5, 14.6, 19.9, 10, 50.2 and 9.5 % of the fasting glucose, insulin, HOMA IR, QUICKI, triglycerides, HDL-cholesterol, SBP Z Score, DBP Z Score, CRP, fibrinogen, adiponectin, leptin and resistin variances.

Adding WHR Z Score improved fasting insulin (R^2^: 45.9 %; r^2^_partial_: 3.9 %), HOMA IR (R^2^: 44.7 %; r^2^_partial_: 3.6 %), QUICKI (R^2^: 43.3 %; r^2^_partial_: 3.3 %), HDL-cholesterol (R^2^: 9.6 %; r^2^_partial_: 5.6 %), triglycerides (R^2^: 11.7 %; r^2^_partial_: 4.2 %), adiponectin (R^2^: 14.3 %; r^2^_partial_: 4.7 %) and CRP (R^2^: 18.2 %.; r^2^_partial_: 4.3 %) prediction.

Associating Waist C Z Score with BMI Z Score, age, sex and pubertal status showed similar findings except for CRP. Indeed, Waist C Z Score accounted for 3.2 % of fasting insulin variance (R^2^: 45.5 %), respectively for 2.6 % of HOMA IR (R^2^: 44.2 %), 2.5 % of QUICKI (R^2^: 42.9 %), 6.8 % of HDL-cholesterol (R^2^: 10.8 %), 4.7 % of triglycerides (R^2^: 12.2 %) and 8.5 % of adiponectin (R^2^: 17.7 %) variances.

Associated with BMI Z Score, age, sex and pubertal status, WHtR accounted for 2.4 % of the HDL-cholesterol variance (R^2^: 6.5 %), respectively for 4.4 % of the SBP Z Score (R^2^: 22.4 %), 3 % of the DBP Z Score (R^2^: 20 %), 10.2 % of the CRP (R^2^: 23.3 %) and 2.4 % of the fibrinogen (R^2^: 21.8 %) variances (Table 3).

**Table 3:** Multivariable anthropometric prediction of cardiovascular risk factors in youths

Dependent variable	Model 1: BMI _Z Score_	Model 2: BMI _Z Score_, WHR _Z Score_			Model 3: BMI _Z Score_, Waist C _Z Score_			Model 4: BMI _Z Score_, WHtR		
	R^2^ model 1	R^2^ model 2	r^2^ partial BMI _Z Score_	r^2^ partial WHR _Z Score_	R^2^ model 3	r^2^ partial BMI _Z Score_	r^2^ partial waist C _Z Score_	R^2^ model 4	r^2^ partial BMI _Z Score_	r^2^ partial WHtR
Fasting glucose	0.074^*^	0.074^*^	0.042^*^	0.000	0.080^*^	0.027^*^	0.007	0.074^*^	0.014	0.000
Fasting insulin^a^	0.437^**^	0.459^**^	0.266^**^	0.039^*^	0.455^**^	0.016	0.032^*^	0.440^**^	0.071^**^	0.005
HOMA IR^a^	0.427^**^	0.447^**^	0.262^**^	0.036^*^	0.442^**^	0.019	0.026^*^	0.430^**^	0.070^**^	0.005
QUICKI	0.414^**^	0.433^**^	0.242^**^	0.033^*^	0.429^**^	0.016	0.025^*^	0.416^**^	0.066^**^	0.004
Triglycerides^a^	0.079^*^	0.117^**^	0.033^*^	0.042^*^	0.122^**^	0.009	0.047^*^	0.095^*^	0.000	0.017
HDL cholesterol^a^	0.043^*^	0.096^*^	0.022^*^	0.056^**^	0.108^**^	0.023^*^	0.068^**^	0.065^*^	0.001	0.024^*^
LDL cholesterol	0.011	0.013	0.002	0.002	0.015	0.005	0.004	0.018	0.009	0.007
SBP Z score	0.188^**^	0.200^**^	0.140^**^	0.014	0.201^**^	0.007	0.016	0.224^**^	0.003	0.044^*^
DBP Z score	0.175^**^	0.184^**^	0.130^**^	0.010	0.176^**^	0.028^*^	0.000	0.200^**^	0.006	0.030^*^
CRP^a^	0.146^**^	0.182^**^	0.113^**^	0.043^*^	0.158^**^	0.005	0.014	0.233^**^	0.004	0.102^**^
Fibrinogen^a^	0.199^**^	0.208^**^	0.116^**^	0.012	0.199^**^	0.021^*^	0.001	0.218^**^	0.006	0.024^*^
Adiponectin^a^	0.100^**^	0.143^**^	0.011	0.047^*^	0.177^**^	0.040^*^	0.085^**^	0.107^**^	0.000	0.007
Leptin^a^	0.502^**^	0.502^**^	0.412^**^	0.001	0.502^**^	0.111^**^	0.001	0.511^**^	0.101^**^	0.019
Resistin^a^	0.095^**^	0.095^*^	0.044^*^	0.000	0.097^*^	0.017	0.002	0.095^*^	0.016	0.000

All models were age, sex and pubertal status adjusted

^*^ *P*-value < 0.05; ^**^ *P*-value < 0.001

^a^Log-transformed variables

Finally, as regards DXA measurements, apart from fasting glucose, LDL cholesterol, fibrinogen and leptin, the DXA prediction of every other cardiometabolic risk factor was improved when the trunk/legs fat mass index was added to total fat mass, as well as after the addition of trunk fat mass to total fat mass (models were adjusted on age, sex and pubertal status) (Table 4).

**Table 4:** Multivariable DXA prediction of cardiovascular risk factors in youths

Dependent variable	Model 1: total fat mass	Model 2: total fat mass, trunk/legs fat mass			Model 3: total fat mass, trunk fat mass		
	R^2^ model 1	R^2^ model 2	r^2^ partial total fat mass	r^2^ partial trunk/legs fat mass	R^2^ model 3	r^2^ partial total fat mass	r^2^ partial trunk fat mass
Fasting glucose	0.058^*^	0.058^*^	0.029^*^	0.000	0.058^*^	0.004	0.000
Fasting insulin^a^	0.376^**^	0.425^**^	0.249^**^	0.078^**^	0.395^**^	0.000	0.030^*^
HOMA IR^a^	0.366^**^	0.412^**^	0.242^**^	0.073^**^	0.383^**^	0.000	0.027^*^
QUICKI	0.349^**^	0.394^**^	0.213^**^	0.069^**^	0.365^**^	0.000	0.025^*^
Triglycerides^a^	0.046	0.095^*^	0.029^*^	0.052^*^	0.075^*^	0.016	0.031^*^
HDL cholesterol^a^	0.015	0.105^**^	0.014	0.091^**^	0.066^*^	0.037^*^	0.051^*^
LDL cholesterol	0.010	0.011	0.005	0.001	0.010	0.001	0.000
SBP Z score	0.201^**^	0.226^**^	0.183^**^	0.032^*^	0.230^**^	0.004	0.037^*^
DBP Z score	0.144^**^	0.178^**^	0.127^**^	0.039^*^	0.181^**^	0.010	0.042^*^
CRP^a^	0.165^**^	0.198^**^	0.164^**^	0.039^*^	0.186^**^	0.001	0.025^*^
Fibrinogen^a^	0.216^**^	0.216^**^	0.158^**^	0.000	0.217^**^	0.018	0.000
Adiponectin^a^	0.077^*^	0.137^**^	0.005	0.065^**^	0.112^**^	0.029^*^	0.038^*^
Leptin^a^	0.575^**^	0.578^**^	0.506^**^	0.005	0.582^**^	0.138^**^	0.015
Resistin^a^	0.100^**^	0.120^**^	0.063^**^	0.022^*^	0.120^**^	0.006	0.023^*^

All models were age, sex and pubertal status adjusted

^*^ *P*-value < 0.05; ^**^ *P*-value < 0.001

^a^Log-transformed variables

## Discussion

Our study clearly showed that, in addition to global overweight and obesity, body fat distribution, as assessed by anthropometry, significantly and independently contributes to the prediction of CV risk factors in overweight and obese youth. Insulin resistance markers, in particular, were more accurately predicted by adding WHR Z Score or Waist C Z Score to BMI Z Score. HDL cholesterol was unanimously more accurately predicted by adding to BMI Z Score one of the three selected anthropometric surrogates for body fat distribution. Triglyceride concentration was more accurately predicted after adding either WHR Z Score or Waist C Z Score to BMI Z Score. Inflammation, as assessed by C-reactive protein, had its prediction improved when WHR Z Score and/or WHtR were added to BMI Z Score. WHtR played a similar role in the case of fibrinogen. WHtR played a role also in blood pressure prediction, after combination with BMI Z Score. Adiponectin concentrations seem to be better approached by combining WHR or Waist C Z Scores with BMI Z Score, while resistin and leptin predictions were not affected by the anthropometric measures for body fat distribution. This was also the case of glucose concentrations, the prediction of which was not affected beyond BMI neither by WHR and Waist C Z Scores nor by WHtR. On the other hand, our findings based on anthropometric measures were in coherence with the associations observed between the aforementioned CV risk factors and DXA combinations: total fat mass and trunk fat mass; respectively total fat mass and trunk/legs fat mass.

Significant relationships linking unfavourable CV profiles to body fat distribution measures, beyond BMI, have been observed in adults since the pioneer work of Vague. Vague pointed out abdominal fat toxicity to be responsible for severe obesities and serious associated prognosis in adults, in opposition to the gynoid shapes which do not expose to similar hazardous health complications [44]. Since that study, several epidemiological investigations in adults showed in particular that, beyond fatness degrees as assessed by BMI, Waist C and/or WHR, measuring upper body fat distribution, were significantly correlated with blood pressure, total serum cholesterol, HDL-cholesterol, triglycerides level and/or serum insulin level [30–33].

However, the scarce published studies in children about the usefulness of adding anthropometric surrogates for body fat distribution to BMI remain controversial. Certain American paediatric studies reported, exactly as is shown in the present study, a significant impact of WHR in addition to BMI, to predict HDL-cholesterol and triglycerides, in youth aged 4–19 years [19, 28]. Gillum [18] also showed an improvement in blood pressure prediction in youths (6–17 y) by adding WHR to BMI. Maffeis et al. [20] showed significant associations between Waist C and Apo lipoproteins, HDL-cholesterol, total/HDL cholesterol ratio, blood pressure, after BMI, age and sex adjustments in prepubertal children aged 3 to 11 years old.

Nevertheless, in 15–16 year-old youths, Lawlor et al. [15] concluded with the superiority of BMI on Waist C in predicting blood pressure, fasting glucose and insulin, triglycerides, LDL and HDL-cholesterol. Only BMI was also highlighted by Garnett et al. to track CV risk between childhood and adolescence [13]. Likewise, with a view to detecting arterial hypertension in 8–10 year-old children, Maximova et al. recommended the measurement of BMI rather than Waist C or WHtR [45]. Gillum et al. [24] showed no significant differences between BMI and WHR for the prediction of CRP in Mexican American children (6–11 y). Similar abilities of BMI-for-age and WHtR were also shown by Freedman et al. [26] for the screening of fasting insulin, blood pressure, triacylglycerol, HDL, LDL and total-to-HDL cholesterol ratio in the Bogalusa Heart Study.

These controversies may be partly explained by the different methodologies applied in the studies. Actually, some studies used continuous data [15, 18–20, 28], while others analysed categorical data [13, 24, 26, 27, 45]. Indeed, using categorical rather than continuous data might result in information loss. The lack of standardized international thresholds to define weight status in children (e.g., for normal-weight versus overweight and obesity) may also impact data interpretations. In the current study, we showed different weight status frequencies according to two definitions suggested in the literature: 64 % of obesity and 36 % of overweight according to the IOTF definition [35, 46] and L,M,S Dutch values [42], respectively 80.8 % of obesity and 19.2 % of overweight according to the WHO definition [47]. The lack of a specific national percentile distribution of anthropometric data in youths appears to be an undeniable issue. That constituted a limitation of the current study. However, thanks to the Dutch L, M, S values provided to us by Dr Van Buuren from the Department of Statistics, Quality of Life, Leiden, Netherlands [42, 43], we were able to develop BMI, Waist C and WHR Z Scores after having checked that Luxembourgish and Dutch paediatric BMI means were similar.

The heterogeneity in the relationships between anthropometry and CV risk factors may also be attributed to the age groups considered in the different studies and/or to the few biological parameters tested. Our study sample was characterized by a broad age range and an exhaustive set of cardiovascular risk factors tested.

The selected nature and relatively small size of our sample, including only overweight and obese subjects, might be a limitation of the current study in that it does not allow the extrapolation of our findings to the general paediatric population. However, as young people who may be at higher risk for CV impairments are mostly the overweight and obese ones, the current findings might widely apply to this high-risk population subgroup.

## Conclusions

In conclusion, combining BMI Z Score with only one anthropometric measure for regional fat (i.e., WHR Z Score, Waist C Z Score and/or WHtR) improves the prediction of the cardiometabolic, inflammatory and/or adipokines profiles amongst youth. These findings might be useful to inform research and clinical activities, and might help public health authorities to implement a more appropriate and cost-effective screening of overweight, obesity and related comorbidities in youth.

## Abbreviations

BMI: Body mass index
WHR: Waist-to-hip ratio
Waist C: Waist circumference
WHtR: Waist-to-height ratio
DXA: Dual energy X-ray absorptiometry
HDL-cholesterol: High-density lipoprotein cholesterol
CV: Cardiovascular
SBP: Systolic blood pressure
DBP: Diastolic blood pressure
LDL-cholesterol: Low-density lipoprotein cholesterol
HOMA IR: Homeostasis model assessment of insulin resistance
QUICKI index: Quantitative insulin sensitivity check index
IOTF: International Obesity Task Force
